# Towards refining Raman spectroscopy-based assessment of bone composition

**DOI:** 10.1038/s41598-020-73559-2

**Published:** 2020-10-07

**Authors:** Furqan A. Shah

**Affiliations:** Department of Biomaterials, Sahlgrenska Academy, University of Gothenburg, Gothenburg, Sweden

**Keywords:** Raman spectroscopy, Bone, Biomedical materials, Biomineralization

## Abstract

Various compositional parameters are derived using intensity ratios and integral area ratios of different spectral peaks and bands in the Raman spectrum of bone. The $\nu$_1_-, $\nu$_2_-,$\nu$_3_-, $\nu$_4_ PO_4_^3−^, and $\nu_{1}$ CO_3_^2−^ bands represent the inorganic phase while amide I, amide III, Proline, Hydroxyproline, Phenylalanine, δ(CH_3_), δ(CH_2_), and $\nu$(C–H) represent the organic phase. Here, using high-resolution Raman spectroscopy, it is demonstrated that all PO_4_^3−^ bands of bone either partially overlap with or are positioned close to spectral contributions from the organic component. Assigned to the organic component, a shoulder at 393 cm^−1^ compromises accurate estimation of $\nu$_2_ PO_4_^3−^ integral area, i.e., phosphate/apatite content, with implications for apatite-to-collagen and carbonate-to-phosphate ratios. Another feature at 621 cm^−1^ may be inaccurately interpreted as $\nu$_4_ PO_4_^3−^ band broadening. In the 1020–1080 cm^−1^ range, the ~ 1047 cm^−1^ $\nu$_3_ PO_4_^3−^ sub-component is obscured by the 1033 cm^−1^ Phenylalanine peak, while the ~ 1076 cm^−1^ $\nu$_3_ PO_4_^3−^ sub-component is masked by the $\nu$_1_ CO_3_^2−^ band. With $\nu$_1_ PO_4_^3−^ peak broadening, $\nu$_2_ PO_4_^3−^ integral area increases exponentially and individual peaks comprising the $\nu$_4_ PO_4_^3−^ band merge together. Therefore, $\nu$_2_ PO_4_^3−^ and $\nu$_4_ PO_4_^3−^ band profiles are sensitive to changes in mineral crystallinity.

## Introduction

Raman spectroscopy is a highly versatile and non-destructive tool for bone composition analysis. Using intensity ratios^1^ and integral area ratios^2^ of different spectral peaks and bands, a variety of Raman metrics are derived in order to describe various compositional parameters of bone. Spectral features assigned as amide I, amide III, Proline (Pro), Hydroxyproline (Hyp), Phenylalanine (Phe), δ(CH_3_), δ(CH_2_), and $\nu$(C–H) are taken as markers of the organic component, i.e., collagen^3–5^, while $\nu$_1_ PO_4_^3−^, $\nu$_2_ PO_4_^3−^, $\nu$_3_ PO_4_^3−^, $\nu$_4_ PO_4_^3−^, and $\nu$_1_ CO_3_^2−^ are the main bands associated with the inorganic component, i.e., apatite^6^. Of the various compositional parameters commonly considered, crystallinity of bone mineral is almost invariably estimated as the reciprocal of the full-width at half-maximum (FWHM) of the $\nu$_1_ PO_4_^3−^ peak, centred at 957–962 cm^−1^, while estimation of the apatite-to-collagen ratio and the carbonate-to-phosphate ratio remain arbitrary. Tissue-level mechanical properties of bone are interpreted from the apatite-to-collagen ratio, while tissue dynamics (i.e., maturation and turnover/remodelling) are interpreted from the carbonate-to-phosphate ratio^1,7^, for example in compromised systemic conditions^8–10^ and at the bone-implant interface^4,11^.

The $\nu$_2_ PO_4_^3−^ and $\nu$_4_ PO_4_^3−^ bands are observed in the 350–650 cm^−1^ spectral range of bone and synthetic hydroxyapatite^12^. However, two discrete bands attributable to type-I collagen in the rat tail tendon are also observed in the same spectral range^13^. CO_3_^2−^ substitution for PO_4_^3−^ influences physical properties including crystallite size^14^, thereby restricting mineral crystallinity to below that of carbonate-free apatites. At ≥ 6.5 wt% CO_3_^2−^, which approximates to one CO_3_^2−^ per unit cell, mineral crystallinity is significantly affected^15^. In a typical Raman spectrum of B-type carbonated apatites, the $\nu$_1_ CO_3_^2−^ mode overlaps the $\nu$_3_ PO_4_^3−^ band^16^. In synthetic B-type carbonated apatites, the $\nu$_3_ PO_4_^3−^ band may be visible up to ~ 3 wt% CO_3_^2−^ but is completely enveloped by the $\nu$_1_ CO_3_^2−^ band in bone^17^, where the CO_3_^2−^ content is much higher (~ 7–9 wt%)^18^. However, superimposition of spectral features associated with the inorganic and the organic components remains largely unreported. Using high-resolution Raman spectroscopy, this work examines the overlap between spectral contributions of the organic and inorganic components of the extracellular matrix in bovine and human bone. Furthermore, a comparative analysis of the $\nu$_1_-_,_ $\nu$_2_-_,_ $\nu$_3_-_,_ and $\nu$_4_ PO_4_^3−^ regions of synthetic hydroxyapatite (HAp) and bone is reported.

## Results

### Spectral overlap between organic and inorganic components of bovine bone

**Figure 1:**
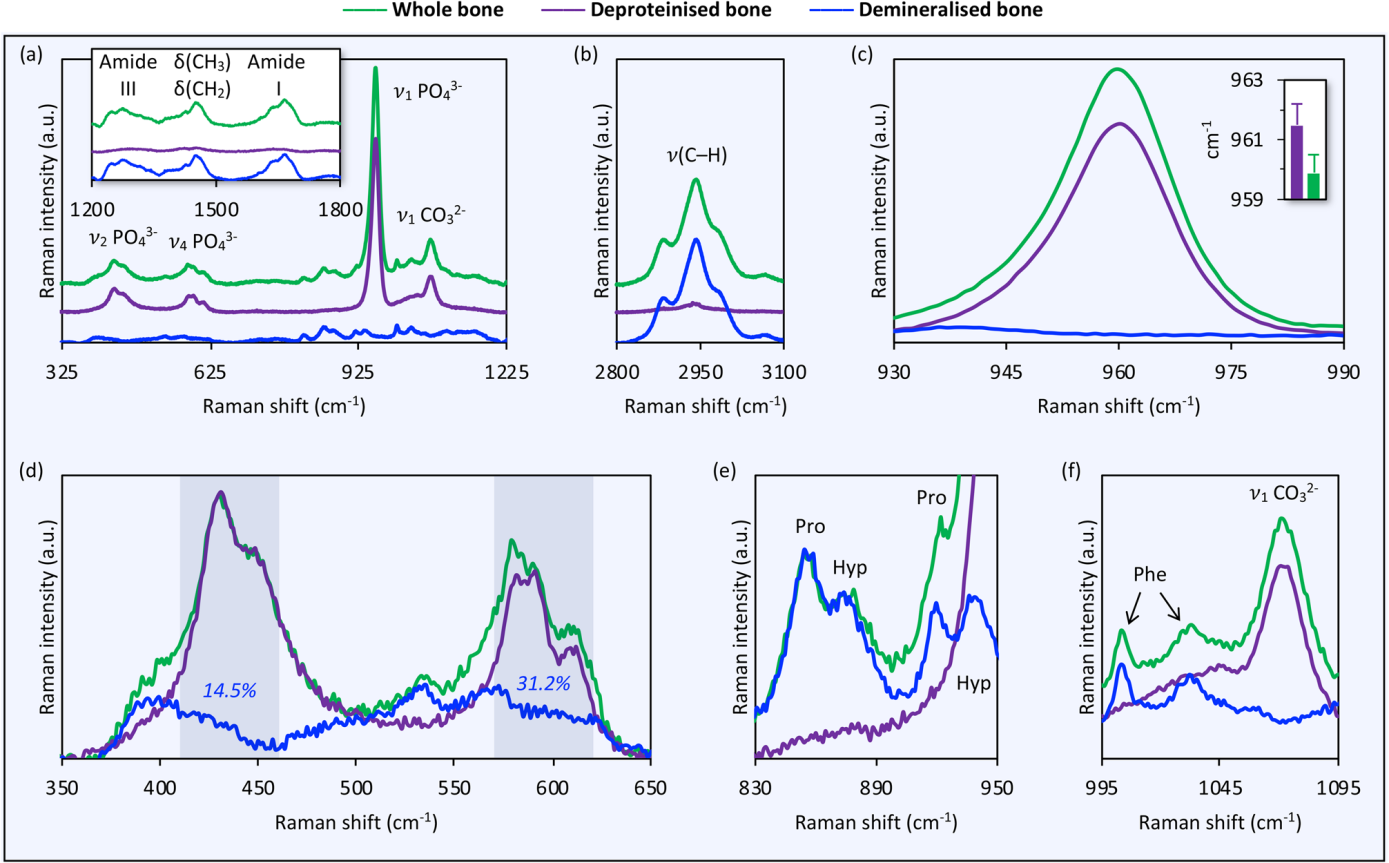
Comparison of whole bone, deproteinised bone, and demineralised bone (averaged Raman spectra, n = 6; 1800 g mm^−1^ grating). (**a**) 325–1225 cm^−1^ range. Inset in (**a**): 1200–1800 cm^−1^ range. (**b**) 2800–3100 cm^−1^ range. (**c**) Demineralisation removes the inorganic phase. Inset in (**c**): $\upnu$_1_ PO_4_^3−^ position (mean values ± standard deviations). (**d**–**f**) Spectral contributions of the organic (demineralised bone) and the inorganic (deproteinised bone) components tend to overlap each other.

Whole bone (Ca/P: 1.45 ± 0.01, N/Ca: 0.6 ± 0.1; in at.%) shows typical spectral features associated with the inorganic and organic components of the extracellular matrix. The organic and inorganic components are isolated by demineralisation using ethylenediaminetetraacetic acid (EDTA) and deproteinisation using sodium hypochlorite (NaOCl), respectively (Supplementary Figure S1). Demineralisation removes the inorganic phase (Ca and P: < 0.01, C: ~ 55, N: ~ 24, and O: ~ 21; in at.%). The $\upnu$_1_ PO_4_^3−^ band, typically the most prominent spectral feature of calcium phosphates and apatite^19^, is no longer visible (Fig. 1). Deproteinisation removes most of the organic component (Ca/P: 1.51 ± 0.01, N/Ca: 0.08 ± 0.05; in at.%), however, minor traces remain detectable. In the 350–650 cm^−1^ range, spectral contributions of the organic component are evident at 390–410 cm^−1^ as a well-defined shoulder on the lower wavenumber side of the $\nu$_2_ PO_4_^3−^ band (380–410 cm^−1^) and at 520–545 cm^−1^. There is considerable overlap between the organic and inorganic components, particularly in the $\nu$_2_ PO_4_^3−^ and $\nu$_4_ PO_4_^3−^ regions. Demineralised bone comprises integral areas equivalent to 14.5% (at 410–460 cm^−1^) and 31.2% (at 570–620 cm^−1^), on average, those of whole bone. Peaks at 920 cm^−1^ and 940 cm^−1^ are attributable to $\nu$(C–C) modes of Pro and Hyp. The 940 cm^−1^ peak is overlapped by the $\nu$_1_ PO_4_^3−^ resulting in a minor broadening of the $\nu$_1_ PO_4_^3−^ band and a small shift towards lower wavenumbers. Distinct features at 1004 cm^−1^ and 1033 cm^−1^ in whole bone and demineralised bone are attributable to Phe.

### FWHM $\upnu$_1_ PO_4_^3−^ correlates with CO_3_^2−^ content

**Figure 2:**
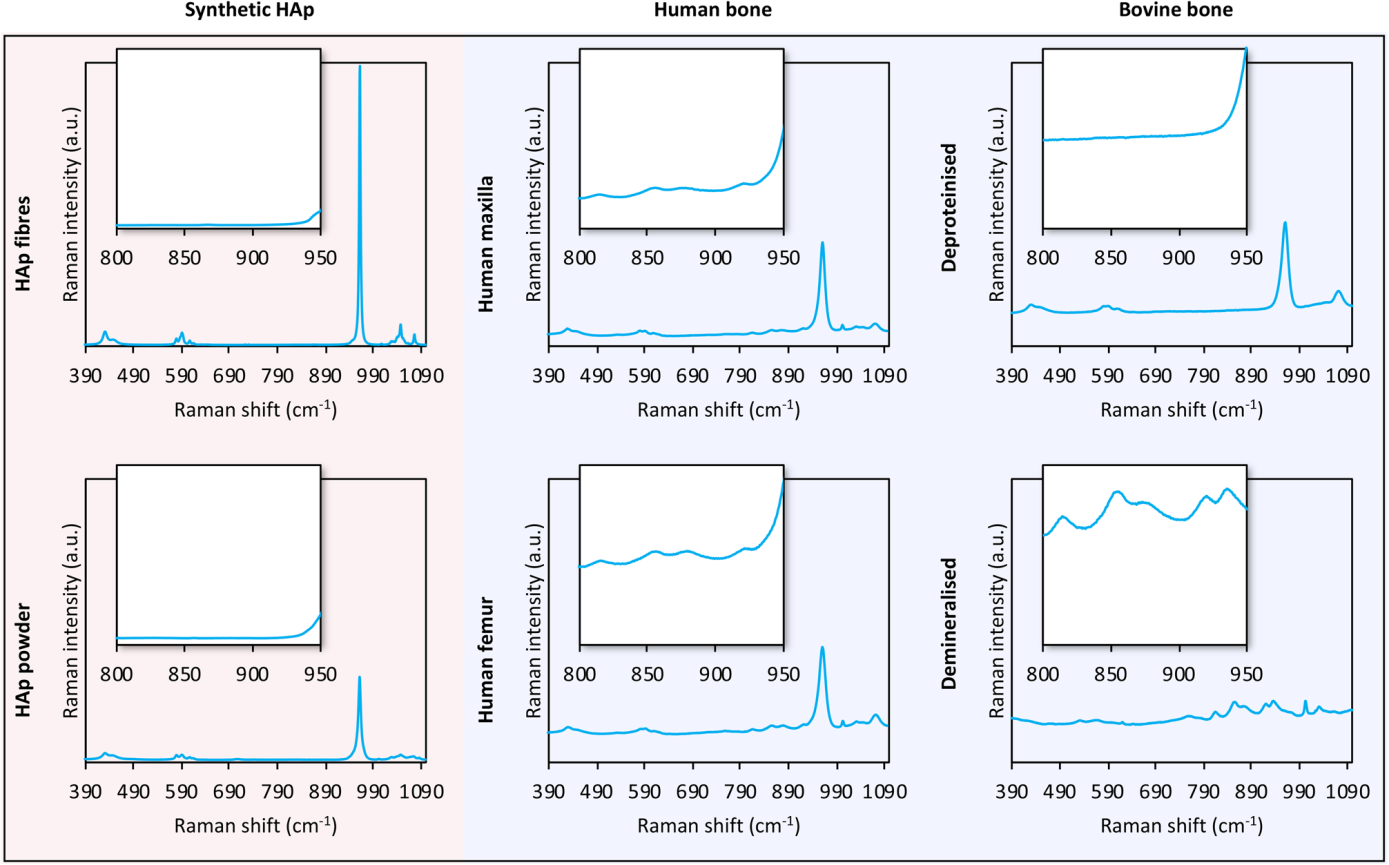
Unprocessed spectra without baseline subtraction and cosmic ray removal (averaged Raman spectra, n = 72; 2400 g mm^−1^ grating). Insets: 800–950 cm^−1^ range (y-axis truncated at 25% height).

**Figure 3:**
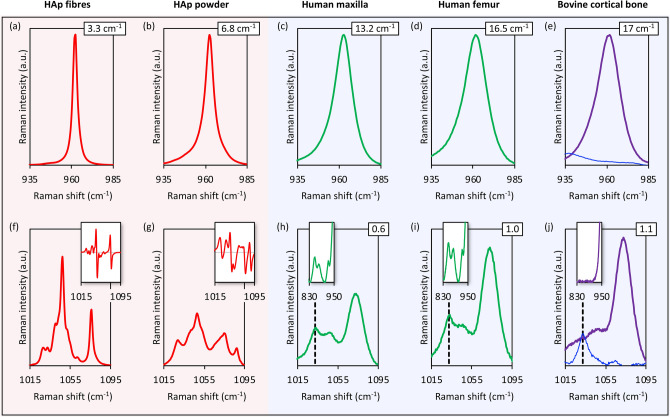
$\upnu$_1_ PO_4_^3−^, $\upnu$_3_ PO_4_^3−^, and $\upnu$_1_ CO_3_^2−^ regions of synthetic HAp and bone (averaged Raman spectra, n = 72; 2400 g mm^−1^ grating). (**a**, **f**) HAp fibres, (**b**, **g**) HAp powder, (**c**, **h**) human maxilla, (**d**, **i**) human femur, and (**e**, **j**) bovine cortical bone (purple = deproteinised; blue = demineralised). (**a**–**e**) The 935–985 cm^−1^ range showing the $\upnu$_1_ PO_4_^3−^ peak. The FWHM $\upnu$_1_ PO_4_^3−^ is indicated. (**f**–**j**) The 1015–1095 cm^−1^ range showing the $\upnu$_3_ PO_4_^3−^ and $\upnu$_1_ CO_3_^2−^ bands. The ν_1_ CO_3_^2−^/ν_2_ PO_4_^3−^ integral area ratio is indicated. The feature at 1033 cm^−1^ (broken lines) is attributable to Phe. Insets in (**f**), (**g**): Second derivative spectra for sub-component identification. Insets in (**h**–**j**): The 830–950 cm^−1^ range showing the $\upnu$(C–C) modes attributable to Pro and Hyp of the organic component.

Compared to synthetic HAp (fibres and powder), human and bovine bone generate markedly higher background fluorescence, which is particularly strong for demineralised bone (Fig. 2). HAp fibres (Ca/P: 1.60 ± 0.10 at.%) show a very intense and narrow $\nu$_1_ PO_4_^3−^ peak (FWHM: 3.3 cm^−1^), while HAp powder (Ca/P: 1.56 ± 0.12 at.%) shows broadening of the $\nu$_1_ PO_4_^3−^ peak (FWHM: 6.8 cm^−1^), indicating differences in mineral crystallinity (Fig. 3). In bone, the $\nu$_1_ PO_4_^3−^ peak is significantly broader (FWHM: 13.25–16.97 cm^−1^) than synthetic HAp and therefore the mineral crystallinity is markedly lower. In addition, a strong ν_1_ CO_3_^2−^ band is centred at ~ 1071 cm^−1^, and only the 1046 cm^−1^ $\nu$_3_ PO_4_^3−^ sub-component can be identified. The FWHM $\nu$_1_ PO_4_^3−^ increases linearly (*r*^2^ = 0.996) with increasing CO_3_^2−^ content (ranging between ~ 0.6 and ~ 1.1), estimated as the integral area ratio of ν_1_ CO_3_^2−^ (~ 1071 ± 15 cm^−1^) to ν_2_ PO_4_^3−^ (410–460 cm^−1^).

### The $\upnu$_3_ PO_4_^3−^ band is overlapped by $\upnu$_1_ CO_3_^2−^ and 1033 cm^−1^ Phe peak

As seen in synthetic HAp, the $\nu$_3_ PO_4_^3−^ band exhibits a number of sub-components between 1020 and 1080 cm^−1^, where the strongest sub-components are found at ~ 1047 cm^−1^ and ~ 1076 cm^−1^. Calculated second derivative spectra reveal fewer $\nu$_3_ PO_4_^3−^ sub-components in HAp powder than HAp fibres (Fig. 3f,g). Whole bone from the human maxilla (Ca/P: 1.3 ± 0.02, N/Ca: 1.25 ± 0.26; in at.%) and human femur (Ca/P: 1.43 ± 0.02, N/Ca: 0.9 ± 0.15; in at.%) and deproteinised bovine bone display a feature consistent with the ~ 1047 cm^−1^ sub-component, however, the ~ 1076 cm^−1^ sub-component is completely masked by the $\nu$_1_ CO_3_^2−^ band. In the 1020–1055 cm^−1^ region, deproteinised bone shows a broad, poorly resolved band that corresponds to ~ 1028–1032 cm^−1^ and ~ 1047 cm^−1^ $\nu$_3_ PO_4_^3−^ sub-components. The 1033 cm^−1^ Phe peak is observed in whole bone (human maxilla and human femur) but not in deproteinised bone.

### $\upnu$_2_ PO_4_^3−^ area increases and $\upnu$_4_ PO_4_^3−^ is poorly resolved with $\upnu$_1_ PO_4_^3−^ peak broadening

**Figure 4:**
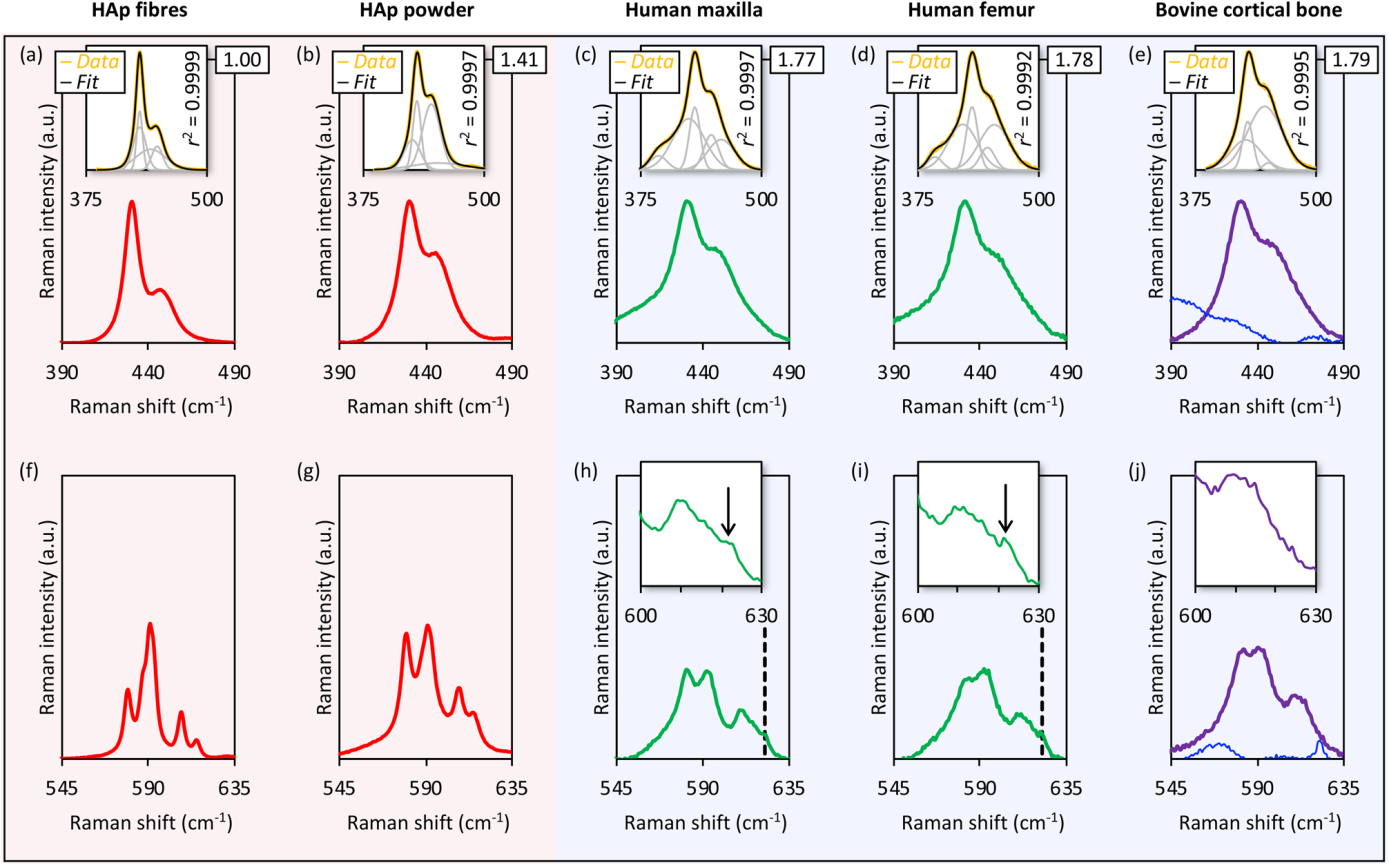
The $\upnu$_2_ PO_4_^3−^ and $\upnu$_4_ PO_4_^3−^ regions of synthetic HAp and bone (averaged Raman spectra, n = 72; 2400 g mm^−1^ grating). (**a**, **f**) HAp fibres, (**b**, **g**) HAp powder, (**c**, **h**) human maxilla, (**d**, **i**) human femur, and (**e**, **j**) bovine cortical bone (purple = deproteinised; blue = demineralised). (**a**–**e**) The 390–490 cm^−1^ range showing the $\upnu$_2_ PO_4_^3−^ band. Relative integral area of the $\upnu$_2_ PO_4_^3−^ band is indicated. Insets in (**a**–**e**): $\upnu$_2_ PO_4_^3−^ band deconvolution by Gaussian curve-fitting of the 375–500 cm^−1^ envelope (yellow line = experimental data; black line = fit). The coefficient of determination (*r*^2^) is indicated. The broad feature at 392 cm^−1^ originates from the organic component. (**f**–**j**) The 545–635 cm^−1^ range showing the $\upnu$_4_ PO_4_^3−^ band. The feature at 621 cm^−1^ (broken lines) is attributable to the organic component. Insets in (**h**–**j**): Detail of the 600–630 cm^−1^ range showing the 621 cm^−1^ shoulder in whole bone (black arrows), which is absent in deproteinised bone.

The $\nu$_2_ PO_4_^3−^ band of synthetic HAp consists of two features at 428 cm^−1^ and 450 cm^−1^ (Fig. 4). The integral area of the $\nu$_2_ PO_4_^3−^ band increases exponentially (*r*^2^ = 0.983) with increasing FWHM $\nu$_1_ PO_4_^3−^. A shift in the relative contributions of the 428 cm^−1^ and 450 cm^−1^ sub-components results in a change in the $\nu$_2_ PO_4_^3−^ band profile. The $\nu$_2_ PO_4_^3−^ band in synthetic HAp and deproteinised bone can be optimally modelled using four Gaussian curves. In the case of bone (human maxilla and human femur), a fifth Gaussian curve centred at 392 cm^−1^ represents the organic component. The $\nu$_4_ PO_4_^3−^ band of synthetic HAp comprises peaks at ~ 580 cm^−1^, 590 cm^−1^, 607 cm^−1^, and 614 cm^−1^. In HAp fibres, the 590 cm^−1^ peak is very strong, with a shoulder at 587 cm^−1^, and approximately twice as intense as the 580 cm^−1^ peak. In HAp powder, though the 580 cm^−1^ and 590 cm^−1^ peaks remain easily distinguishable, their intensities are nearly similar. In bone (whole and deproteinised), splitting of the 580 cm^−1^ and 590 cm^−1^ peaks is minimal, while the 607 cm^−1^, and 614 cm^−1^ peaks completely merge together. A shoulder is observed at ~ 621 cm^−1^ in whole bone (human maxilla and human femur), but not in deproteinised bone.

## Discussion

Using high-resolution Raman spectroscopy, this work explores the overlap between the spectral features assigned to the organic and inorganic components of the extracellular matrix. Furthermore, a comparative analysis of the $\nu$_1_-_,_ $\nu$_2_-_,_ $\nu$_3_-_,_ and $\nu$_4_ PO_4_^3−^ bands is presented for two types of synthetic HAp varying in crystallinity, human bone obtained from two anatomical sites (the maxilla and the femur), and the inorganic component of bovine cortical bone. The spectral profile (incl. peak positions and band sub-components) of high-temperature sintered and highly-oriented HAp fibres closely matches that of human bone heated to 900 °C^20^. The HAp powder is polycrystalline^21^. The use of human maxilla and human femur, here, only represents two different anatomical sites presumably having widely divergent biological characteristics (tissue age, microstructure, biomechanical properties etc.) rather than a direct comparison between them.

Raman spectroscopy of biological materials is particularly prone to the presence of a background fluorescence signal^22^. Compared to green lasers (e.g., 532 nm), the fluorescence generated by biological tissues can be efficiently suppressed using a red laser (e.g., 633 nm)^23^. Additionally, spectral baselines affected by background fluorescence can be corrected by polynomial baseline fitting^24^. Here, human and bovine bone generated stronger background fluorescence than synthetic HAp. Demineralised bovine bone, despite exhibiting the most intense background fluorescence, displayed many of the characteristic spectral features associated with type-I collagen^13,25,26^, both before and after baseline subtraction.

Formalin fixation reduces Raman signal intensity compared to fresh (i.e., unfixed) tissue, however, soaking in aqueous media such as phosphate-buffered saline can significantly reverse such effects^27^. Here, human bone (exposed to formalin) and bovine bone (not exposed to formalin) display comparable Raman spectral characteristics in the context of the overlap between the organic and inorganic components. While long-term storage of bone in formaldehyde may induce various compositional alterations^28^, Raman bone quality parameters remain largely unaffected by short-term (~ 12 h) exposure to 4% paraformaldehyde although 3–4% decrease in collagen maturity (ratio between amide I sub-components at 1660 cm^−1^ and 1690 cm^−1^) has been reported^29^.

The data indicate that all of the PO_4_^3−^ bands in a typical Raman spectrum of bone either partially overlap with or are in close proximity to spectral contributions from the organic component. Most notably, the shoulder at 393 cm^−1^ encroaches upon the $\nu$_2_ PO_4_^3−^ band of bone, and may affect the accuracy of phosphate/apatite content estimation in more recently formed tissue, e.g., at the mineralisation front. Therefore, the contribution of this feature must be taken into consideration and ideally subtracted. The shoulder on the higher wavenumber side of the $\nu$_4_ PO_4_^3−^ band (at ~ 621 cm^−1^) assigned to the organic component can be inaccurately interpreted as $\nu$_4_ PO_4_^3−^ band broadening, particularly where measurements are obtained at lower spectral resolution (e.g., 600 g mm^−1^ grating) or at shorter acquisition times.

The Hyp peak at 940 cm^−1^ lies in close proximity to the $\nu$_1_ PO_4_^3−^ band, and may induce an apparent shift of the $\nu_{1}$ PO_4_^3−^ peak position to lower wavenumbers in raw experimental data, as seen for deproteinised bovine bone ($\nu$_1_ PO_4_^3−^ at 961.5 cm^−1^) vs. whole bone ($\nu$_1_ PO_4_^3−^ at 959.9 cm^−1^). The slightly elevated Ca/P ratio of deproteinised bone is likely related to the loss of non-collagenous phosphoproteins from the extracellular matrix^30^, rather than higher mineral density. A similar shift of the $\nu$_1_ PO_4_^3−^ peak position to lower wavenumbers is observed for the human bone specimens, where the organic content of the maxilla ($\nu$_1_ PO_4_^3−^ at 962.8 cm^−1^) is lower than that of the femur ($\nu$_1_ PO_4_^3−^ at 961.4 cm^−1^).

In the Raman spectrum of bone, the ~ 1047 cm^−1^ $\nu$_3_ PO_4_^3−^ sub-component is superimposed on a broad, poorly resolved band. Owing to the presence of the 1033 cm^−1^ Phe peak, identification of the 1047 cm^−1^ $\nu$_3_ PO_4_^3−^ sub-component is challenging, and may be possible only with the use of very high groove density diffraction gratings (e.g., 2400 g mm^−1^ or better). In deproteinised bone, the 1033 cm^−1^ Phe peak is absent while the broad 1025–1055 cm^−1^ band is conserved, confirming that the latter indeed arises from the inorganic component. Furthermore, it is likely that at least some $\nu$_3_ PO_4_^3−^ sub-components are overlapped by the $\nu_{1}$ CO_3_^2−^ band, in agreement with previous reports suggesting that the 1076 cm^−1^ $\nu$_3_ PO_4_^3−^ sub-component is masked by the $\nu_{1}$ CO_3_^2−^ band^31^.

Spectral features at 380–410 cm^−1^, 520–545 cm^−1^, 940 cm^−1^, and 1033 cm^−1^, are also notably absent for bioapatite formed without a collagenous extracellular matrix, e.g., in a mineralised bacterial biofilm^32^, further confirming that these features originate from the organic component of bone. Extraction of the organic component using hot (118 °C) ethylenediamine for ~ 20–30 distillation cycles efficiently reduces the nitrogen content of bone to below 0.1%^33^. However, minor traces of the organic component are difficult to eliminate at lower temperatures, even with the combination of enzymatic (lipase and collagenase) degradation at 37 °C followed by NaOCl exposure at room temperature and drying at 110 °C^34^. An explanation for this may be the inherent nanometre-scale arrangement of bone where polycrystalline plates of extrafibrillar mineral (referred to as ‘mineral lamellae’) closely wrap around single collagen fibrils^33,35,36^, forming an organic–inorganic core–shell structure^37^.

It has been estimated from X-ray diffraction measurements and Raman spectroscopy that the crystallite size of carbonated apatite decreases from 86 ± 6 nm (0.3 wt% CO_3_^2−^) to 27.5 ± 0.8 nm (8.6 wt% CO_3_^2−^) which correspond to FWHM $\nu$_1_ PO_4_^3−^ of 7.3 cm^−1^ and 16.4 cm^−1^, respectively^15^. Besides the degree of atomic order^19^, the line widths of stretching ($\nu$) Raman modes are sensitive to the local crystal field^38^. Thus in comparison to HAp fibres^39^, $\nu$_1_ PO_4_^3−^ peak broadening observed for the HAp powder is additionally attributable to optical phonon confinement effects in nanocrystals^40^.

The $\nu$_2_ PO_4_^3−^ and $\nu$_4_ PO_4_^3−^ band profiles also provide valuable information regarding mineral crystallinity. The data suggest that simultaneous with $\nu$_1_ PO_4_^3−^ peak broadening (i.e., decreasing mineral crystallinity), the $\nu_{2}$ PO_4_^3−^ band area increases while individual peaks comprising the $\nu$_4_ PO_4_^3−^ band become progressively less distinct. A similar trend in $\nu$_2_ PO_4_^3−^ band broadening has been reported for bone and dentine compared to dental enamel^16^, where the apatite crystallites are much larger in size^41^. Across the different bone specimens investigated here, deviations in FWHM $\nu$_1_ PO_4_^3−^ (~ 29%) and $\nu$_2_ PO_4_^3−^ integral area (~ 1.1%) are relatively small despite a large change in CO_3_^2−^ content (~ 81%). This trend is consistent with previous reports that B-type CO_3_^2−^ substitution minimally affects apatite lattice parameters^42^. Since bone mineral is essentially ion substituted, poorly crystalline apatite^43^, where physical constraints of the collagen network^44,45^ and presence of citrate ions^46^ are at play, it is likely that CO_3_^2−^ incorporation plays only a limited role in restricting crystallite size. Substantial B-type CO_3_^2−^ substitution may be required in order to induce a significant, further impact on bone mineral crystallinity.

## Conclusions

In comparison to synthetic hydroxyapatite, bone generates stronger background fluorescence, which can be efficiently suppressed through optimisation of Raman spectral acquisition conditions/parameters. In a typical Raman spectrum of bone, all PO_4_^3−^ bands ($\nu$_1_-_,_ $\nu$_2_-_,_ $\nu$_3_-_,_ and $\nu$_4_ PO_4_^3−^) either partially overlap with or are in close proximity to spectral contributions from the organic component. Therefore, to avoid misleading quantification of apatite-to-collagen and carbonate-to-phosphate ratios, the upper and lower wavenumber limits warrant careful consideration when PO_4_^3−^ integral areas are estimated. A broad shoulder at 393 cm^−1^ may compromise accurate estimation of phosphate/apatite content. Another feature at 621 cm^−1^ may be inaccurately interpreted as $\nu$_4_ PO_4_^3−^ band broadening. The ~ 1047 cm^−1^ $\nu$_3_ PO_4_^3−^ sub-component is partially obscured by the 1033 cm^−1^ Phe peak, while the ~ 1076 cm^−1^ $\nu$_3_ PO_4_^3−^ sub-component is masked by the $\nu$_1_ CO_3_^2−^ band. $\nu$_2_ PO_4_^3−^ and $\nu$_4_ PO_4_^3−^ band profiles vary with mineral crystallinity. Concurrent with $\nu$_1_ PO_4_^3−^ peak broadening, the $\nu$_2_ PO_4_^3−^ band area increases and individual peaks comprising the $\nu$_4_ PO_4_^3−^ band become progressively less distinct. To this end, an analytical approach involving deproteinisation and/or demineralisation may be pertinent, e.g., in the investigation of forensic and archaeological (human and faunal) bone, which may contain biological contaminants or reveal diagenetic alterations.

## Methods

### Bovine bone

Bovine cortical bone discs (400 µm thick) stored in 96% ethanol (https://boneslices.com) were either (1) deproteinised, i.e., isolated inorganic component, using 5% sodium hypochlorite (NaOCl; 1 mL, 2 × 8 h at 4 °C)^47^, (2) demineralised, i.e., isolated organic component, using 10% ethylenediaminetetraacetic acid in 0.1 M tris(hydroxymethyl)aminomethane and 7.5% polyvinylpyrrolidone (EDTA; 1 mL, 2 × 24 h at 4 °C)^48^, or (3) used in the whole/native state. In comparison to whole bone, deproteinised bone is embrittled and chalky white in appearance while deproteinised bone is flexible, semi-transparent, membrane-like, and highly sensitive to dehydration. Deproteinised, demineralised, and whole bone discs were rinsed (× 3) with Hank’s Balanced Salt Solution (Gibco) and subsequently maintained in a hydrated state at 4 °C, but allowed to dry at room temperature (~ 3–5 min) immediately prior to the analytical procedures.

### Human bone

Specimens of human bone were obtained from the human maxilla during elective removal of a dental implant and from the femur during elective removal of a bone-anchored amputation prosthesis using trephine drills. Bone specimens were transported in 10% neutral buffered formalin at 4 °C, rinsed (× 3) with Hank’s Balanced Salt Solution (Gibco) and subsequently maintained in a hydrated state at 4 °C, but allowed to dry at room temperature (~ 3–5 min) immediately prior to the analytical procedures. Informed consent was obtained from both subjects. The study protocol was approved by the Regional Ethical Review Board of Gothenburg (Dnr 434-09 and Dnr 130-09). All experiments were performed in accordance with relevant guidelines and regulations.

### Synthetic HAp

HAp fibres were produced by dispersing nanometre-sized (5–80 nm) apatite particles in an aqueous solution of pullulan (200 kDa), followed by simultaneously extruding this solution under pressure and discharging air at high speed (250 m/s) to form a stream of fibres. The fibre stream was heated at 400 °C using a far-infrared heater positioned under the extrusion nozzles and then blown onto a screen conveyor belt. A non-woven fabric thus collected was further heated at 50 °C per h and calcined at 1100 °C for 1 h^49^. HAp powder (< 200 nm particle size, CAS 12167-74-7, Sigma Aldrich, Product No. 677418) having an average crystallite size of ~ 85 nm^50^ was commercially sourced.

### Energy dispersive X-ray spectroscopy

Elemental analysis was performed using energy dispersive X-ray spectroscopy (EDX; INCA EDX system, Oxford Instruments GmbH, Wiesbaden, Germany) in a Quanta 200 environmental scanning electron microscope (FEI Company, The Netherlands) operated. To estimate the Ca, P, O, C, and N content of synthetic HAp and bone, eight locations were analysed at 0.5 Torr water vapour pressure, 20 kV accelerating voltage, 0–10 keV spectral energy range, and 10 mm working distance.

### Micro-Raman spectroscopy

Micro-Raman spectroscopy was performed using a confocal Raman microscope (Renishaw inVia Qontor) equipped with a 633 nm laser and LiveTrack focus-tracking technology. The laser was focused down on to the sample surface using a × 100 (0.9 NA) objective^39^. The Raman scattered light was collected using a Peltier-cooled charge-coupled device deep depletion near-infrared enhanced detector behind an 1800 g mm^−1^ grating (step size of 1.0 ± 0.15 cm^−1^; SynchroScan wide-range scanning mode; ~ 300 cm^−1^ to 3200 cm^−1^ spectral range; ~ 60 s integration time and 5 accumulations per spectrum) or a 2400 g mm^−1^ (step size of 0.75 ± 0.04 cm^−1^; ~ 350 cm^−1^ to ~ 1100 cm^−1^ spectral range; 10 s integration time and 10 accumulations per spectrum) grating. The laser power at the sample was ~ 15 mW. In Renishaw WiRE 5.2 software, background fluorescence removal was performed using *intelligent polynomial* fitting baseline subtraction (11th order) followed by cosmic ray removal.

## Supplementary information

Supplementary information.
