# Supplementary material for: Towards refining Raman spectroscopy-based assessment of bone composition
Source: Sci Rep. 2020 Oct 7;10:16662. doi: 10.1038/s41598-020-73559-2 (PMC7541616; doi:10.1038/s41598-020-73559-2)
Supplement: Supplementary file 1 — Supplementary information. [file 41598_2020_73559_MOESM1_ESM.pdf]

SUPPLEMENTARY INFORMATION

Towards refining Raman spectroscopy-based assessment of bone composition

Furqan A. Shah

Department of Biomaterials, Sahlgrenska Academy, University of Gothenburg, Gothenburg, Sweden

Corresponding author:

Furqan A. Shah

https://orcid.org/0000-0002-9876-0467

Email: furqan.ali.shah@biomaterials.gu.se

Tel: +46 31 786 28 98

Deproteinisation using 5% sodium hypochlorite (NaOCl) and demineralisation using 10% ethylenediaminetetraacetic acid (EDTA) can be monitored using micro-Raman spectroscopy.

Micro-Raman spectroscopy was performed using a confocal Raman microscope (Renishaw inVia Qontor) equipped with a 633 nm laser and LiveTrack focus-tracking technology. The laser was focused down on to the sample surface using a ×100 (0.9 NA) objective. The Raman scattered light was collected using a Peltier-cooled charge-coupled device deep depletion near-infrared enhanced detector behind an 1800 g mm^{-1} grating (step size of 1.0 ± 0.15 cm^{-1}; SynchroScan wide-range scanning mode; ~100 cm^{-1} to 3200 cm^{-1} spectral range; ~70 s integration time and 3 accumulations per spectrum). The laser power at the sample was ~15 mW. In Renishaw WiRE 5.2 software, background fluorescence removal was performed using *intelligent polynomial* fitting baseline subtraction (11th order) followed by cosmic ray removal.

Here, elimination of organic constituents was evaluated after 2 h and 6 h exposure to 5% NaOCl **(Fig. S1 a–c)**, and the process was continued until 16 h. Likewise, removal of inorganic constituents was evaluated after 6 h and 24 h exposure to 10% EDTA **(Fig. S1 d–f)**, and the process was continued until 48 h. No further change is observed beyond 16 h exposure to 5% NaOCl or 48 h exposure to 10% EDTA in the respective Raman spectra of deproteinised and demineralised bone.

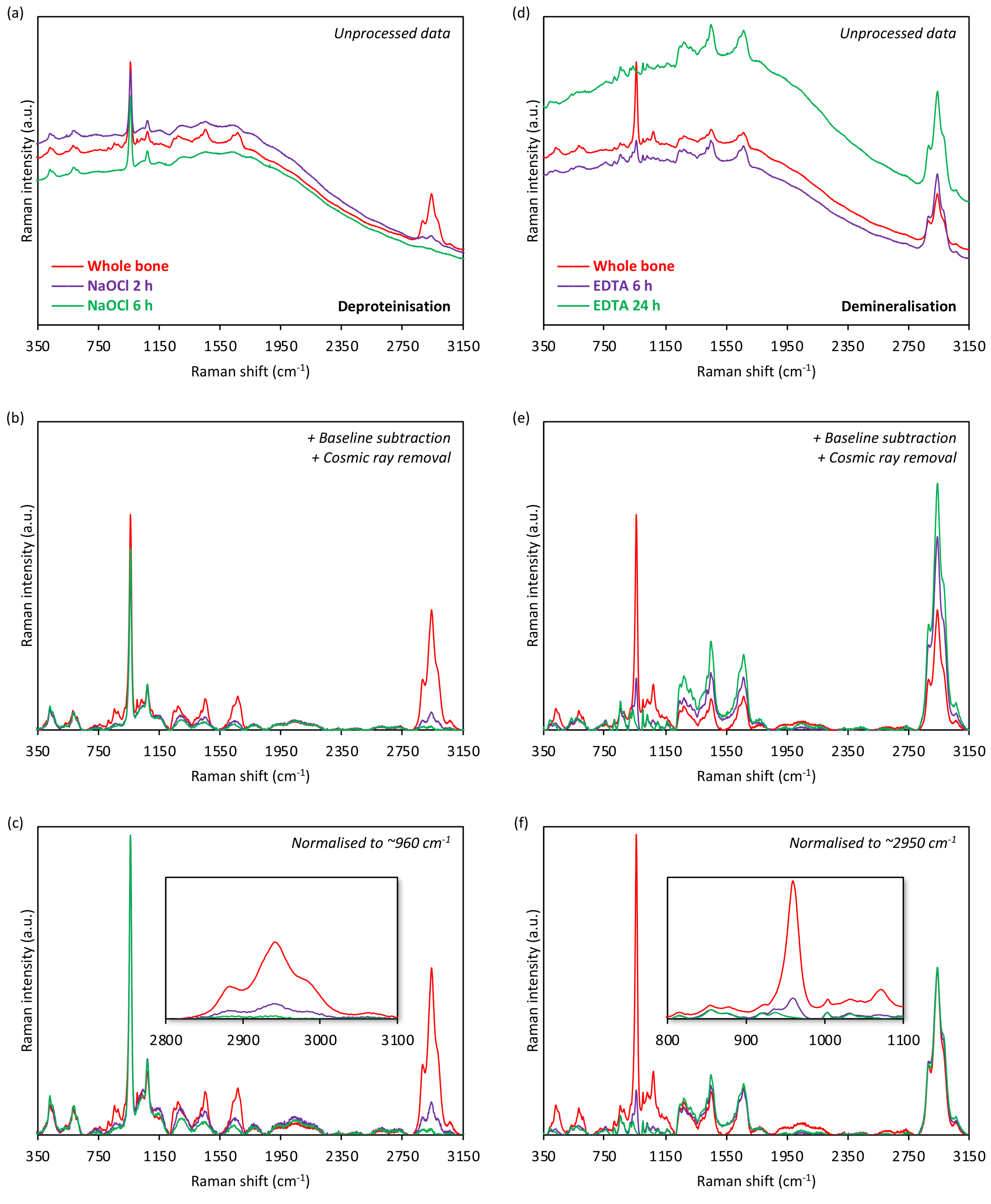

Fig. S1:

Monitoring deproteinisation and demineralisation (averaged Raman spectra, n = 3; 1800 g mm^{-1} grating). (**a–c**) Upon exposure to 5% NaOCl, spectral features attributed to amide I, amide III, Proline, Hydroxyproline, Phenylalanine, δ(CH_3), δ(CH_2), and ν(C–H) decrease as a function of time. Inset in (**c**): Detail of the 2800–3100 cm^{-1} range. (**d–f**) Upon exposure to 10% EDTA, the ν_1 PO_4^{3-} peak, and ν_2 PO_4^{3-}, ν_4 PO_4^{3-}, and ν_1 CO_3^{2-} bands are reduced in intensity as a function of time. Inset in (**f**): Detail of the 800–1100 cm^{-1} range.
